# Anticonvulsant and ameliorative effects of pioglitazone on cognitive deficits, inflammation and apoptosis in the hippocampus of rat pups exposed to febrile seizure

**DOI:** 10.22038/ijbms.2019.35056.8339

**Published:** 2019-03

**Authors:** Hussein Allawi Hussein, Ali Moghimi, Ali Roohbakhsh

**Affiliations:** 1 Rayan Center for Neuroscience and Behavior, Department of Biology, Faculty of Science, Ferdowsi University of Mashhad, Iran; 2 Pharmaceutical Research Center, Pharmaceutical Technology Institute, Mashhad University of Medical Sciences, Mashhad, Iran

**Keywords:** Apoptosis, Febrile seizure, Hippocampus, Inflammation, Memory, Pioglitazone

## Abstract

**Objective(s):**

Pioglitazone (PGZ), a peroxisome proliferator-activated receptor gamma (PPAR-γ) agonist, has significant neuroprotective effects and has been reported to regulate inflammatory processes.

**Materials and Methods:**

We evaluated the effects of PGZ on febrile seizure (FS) in rat pups. Three groups of male rat pups received intraperitoneal (IP) injections of PGZ (5, 10, and 20 mg/kg). Lipopolysaccharide (LPS) and kainic acid (KA) were injected to induce FS. The rat pups behaviors were recorded and analyzed. Seizure latency, duration, and severity were recorded to evaluate the effect of PGZ on FS. Novel object recognition task (NORT) was used to evaluate the effect of PGZ on cognitive deficits induced by FS. At the end of the experimental protocol, molecular and histological tests were done.

**Results:**

PGZ significantly increased seizure latency and decreased seizure duration and median of seizure scores (*P*<0.05, *P*<0.01, and *P*<0.001) after induction of FS. Rat pups exposed to FS had memory deficits both in short-term and long-term memories in the NORT that were reversed by PGZ-treatment (*P*<0.01 and *P*<0.001). PGZ significantly reduced interleukin-1β, tumor necrosis factor-α, and inducible nitric oxide synthase concentration in the hippocampus (*P*<0.05 and *P*<0.01). In addition, PGZ decreased the number of degenerating and TUNEL positive neurons in CA1, CA3, and DG subfields of the hippocampus (*P*<0.05, *P*<0.01 and *P*<0.001).

**Conclusion:**

The present results indicated that PGZ had anticonvulsant, anti-inflammatory, and anti-apoptotic effects with ameliorative effects on cognitive deficits induced by FS in rat pups.

## Introduction

Febrile seizure (FS) is the most prevalent seizure in children aged from 6 months to 5 years (1, 2). Prolonged FS has both acute and long-lasting effects on the developing brain (3). Inflammation is considered to be a key element of the pathophysiology of epilepsy and febrile seizure (4). Therefore, anti-inflammatory drugs retain significant anticonvulsant properties both in experimental and clinical settings (5).

Inflammatory mediators, which are known as triggers of fever, have also been implicated in the onset of seizure attacks (6). During fever, brain temperature is elevated, at least in part, via the release of inflammatory mediators such as cytokines (7, 8). Pro-inflammatory cytokines including interleukin-1β (IL-1β), tumor necrosis factor-α (TNF-α), and IL-10, as an anti-inflammatory cytokine, have been implicated in the initiation and propagation of seizures (9). IL-1β increases N-methyl-D-aspartate (NMDA) receptor activity via activation of tyrosine kinases and subsequent NR2A/B subunit phosphorylation and eventually provokes glutamate-mediated neurodegeneration (10). TNF-α also modulates glutamate receptor trafficking via TNF receptor 1 to increase excitatory synaptic transmission and to induce acute seizures (11). Moreover, inducible nitric oxide synthase (iNOS) is believed to have a fundamental role in the inflammatory processes. It is induced by pro-inflammatory stimuli such as lipopolysaccharide (LPS) or various cytokines (12). Overexpression of iNOS leads to production of nitric oxide (NO), as a cytotoxic mediator, in large amounts and for long periods (13, 14). In addition, NO may be converted to a number of reactive derivatives such as peroxynitrite, NO_2_, N_2_O_3_, and S-nitrosothiols, which can kill neuronal cells by triggering apoptosis (13).

Prolonged FS has been reported with changes in hippocampal synaptic plasticity and may impair long-term memory (15). FS can be provoked by using a combination of LPS and kainic acid (KA) (16, 17). Injection of KA, as an analogue of glutamate, evokes seizures that are accompanied by nerve cell damage primarily in the limbic system (18). 

A target that has been reported to be involved in neuroinflammation is peroxisome proliferator-activated receptor-γ (PPAR-γ) (19). Previous studies demonstrated that activation of PPAR-γ using thiazolidinediones (TZDs) prevented neurodegeneration by decreasing neuroinflammation, improving mitochondrial function, and reducing neuronal death. Pioglitazone is an anti-diabetic drug from the TZDs family and acts as an agonist for PPAR-**γ** (20). Activation of PPAR-**γ** by pioglitazone regulated inflammation and protected neurons against LPS insult at least via inhibiting iNOS expression and NO generation (21). Moreover, pioglitazone via suppression of TNF-α and IL-1β expression induced a survival improving effect on mortality of the septic mice (22). On the other hand, pioglitazone has been reported with significant antiepileptic effects in PTZ-induced seizures in mice. This effect of pioglitazone has been attributed to modulation of NO synthesis (23) and prevention of inflammation and apoptosis (24). So, the aim of the present study was to determine if pioglitazone ameliorates seizure and cognitive deficits induced by FS and to determine the underlying protective mechanisms. 

## Materials and Methods

***Chemicals***

*Escherichia coli* LPS (serotype O26:B6) and KA were purchased from Sigma-Aldrich (USA) and Tocris (UK), respectively. PGZ was a gift from Samisaz Pharmaceutical (Iran) and was dissolved in sterile saline 0.9%. *In situ* cell death detection kit (fluorescein) was purchased from Roche (Germany). Rat IL-1β and TNF-α ELISA kits were purchased from Abcam (USA), and iNOS ELISA kit was purchased from Mybiosource (USA). 

***Animals***

Pregnant female Wistar rats were maintained in the animal house of Faculty of Sciences, Ferdowsi University of Mashhad, under standard environmental conditions (12:12 hrs. of light/dark cycle, 22+2 °C, food and water available *ad libitum*). Rats were monitored daily for the parturition day, which was taken as day 0 (P0). All experimental and animal handling procedures were carried out in accordance with animal care guidelines and were approved ethically by the Ethics Committee for Human and Animal Care of Ferdowsi University of Mashhad.

***Febrile seizure induction***

At day 7 (P7), male rat pups were separated from their dams and placed in cages holding 3–4 pups. FS was induced as reported in previous studies (16, 25). In brief, rats were given intraperitoneal (IP) injections of LPS (200 *μ*g/kg). Rectal body temperatures were recorded by a digital multimeter with 1 hr. intervals till 2.5 hrs., after that, during the increase of fever, rat pups received a sub-convulsive dose of KA (1.75 mg/kg, IP). The LPS injections were given between 9:00 and 10:00 a.m., while KA injections were given between 12:00 and 13:00 p.m. The doses for LPS and KA were chosen based on previous studies (16, 17, 25). The rat pups behaviors were videotaped for 1 hr. and videos were analyzed later by a trained observer. Seizure-related behaviors were rated on the following scale: rat pups exposing only wet dog shakes were rated as stage **1**; those who showed chewing, head bobbing, and forelimb clonus were rated as stage **2**; rats with generalized seizures and rearing were rated as stage **3**; rats with generalized seizures, rearing, and falling (loss of postural tone) were considered as stage **4**; and rat pups that died during seizure were scored as stage **5 **(26, 27).

**Figure 1:**
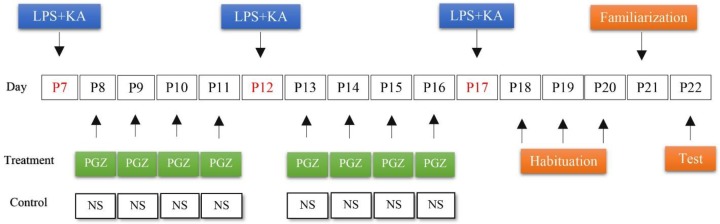
A schematic diagram of experimental protocol. P: Post neonatal day; LPS: Lipopolysaccharide; KA: Kainic acid; PGZ: Pioglitazone; NS: Normal saline

 Seizure latency, the time from injection of LPS and KA to the onset of seizure, and seizure duration, the time to recovery from seizure onset, were recorded (28). Seizure severity was evaluated by calculating the median of seizure scores after induction of febrile seizure (29). Febrile seizure was also repeated later at days 12 and 17 (See Figure 1 for details). 

***Experimental protocol***

Male rat pups were divided into four experimental groups. At day 8 (P8), rat pups were treated with three different doses of PGZ (5, 10, and 20 mg/kg, IP) till day 12 (P12). They also received pioglitazone from day 13 (P13) till day 17 (P17). The pioglitazone injections were given between 9:00 and 11:00 a.m. The control group was given just normal saline (Figure 1).

**Figure 2:**
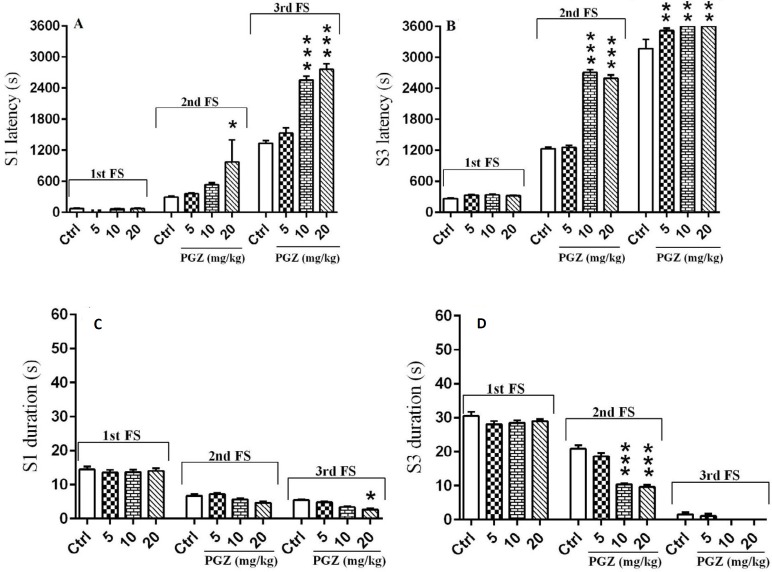
Effect of PGZ (5, 10, and 20 mg/kg) on stage 1 (A) stage 3 seizure latencies (B) stage 1 (C) and stage 3 (D) seizure durations after induction of FS (2nd and 3rd) in rat pups. n=10. *: *P*<0.05, **: *P*<0.01 and ***: *P*<0.001 different from control group. FS: Febrile seizure; S: Stage; Ctrl: Control; PGZ: Pioglitazone

**Figure 3:**
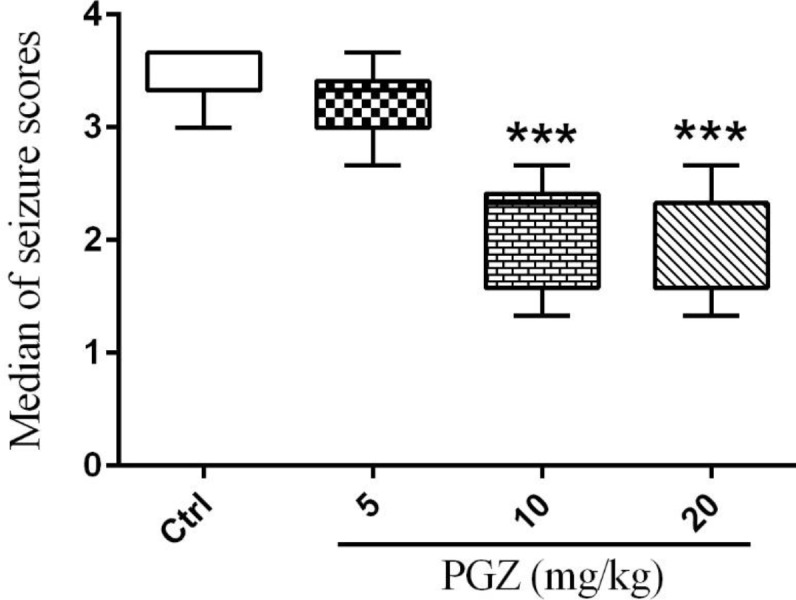
Effect of PGZ (5, 10, and 20 mg/kg) on seizure severity after induction of FS in rat pups. n=10. ***: *P*< 0.001 different from control group. Ctrl: Control; PGZ: Pioglitazone

**Figure 4:**
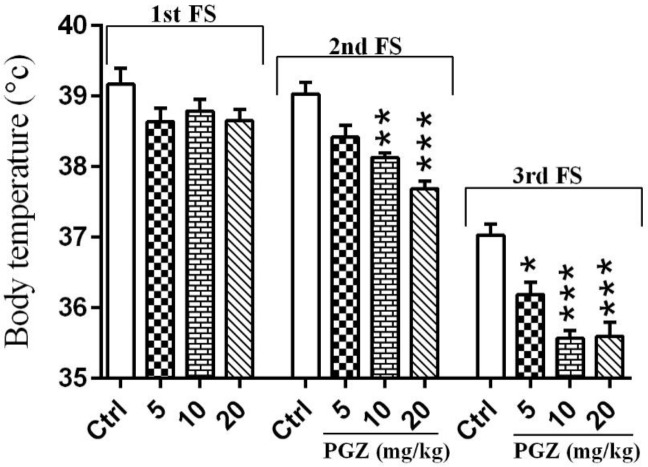
Effect of PGZ (5, 10, and 20 mg/kg) on body temperature of rat pups after induction of FS (2nd, and 3rd). n=10. *: *P*<0.05, **: *P*<0.01 and ***: *P*<0.001 different from control group. FS: Febrile seizure; Ctrl: Control; PGZ: Pioglitazone

**Figure 5:**
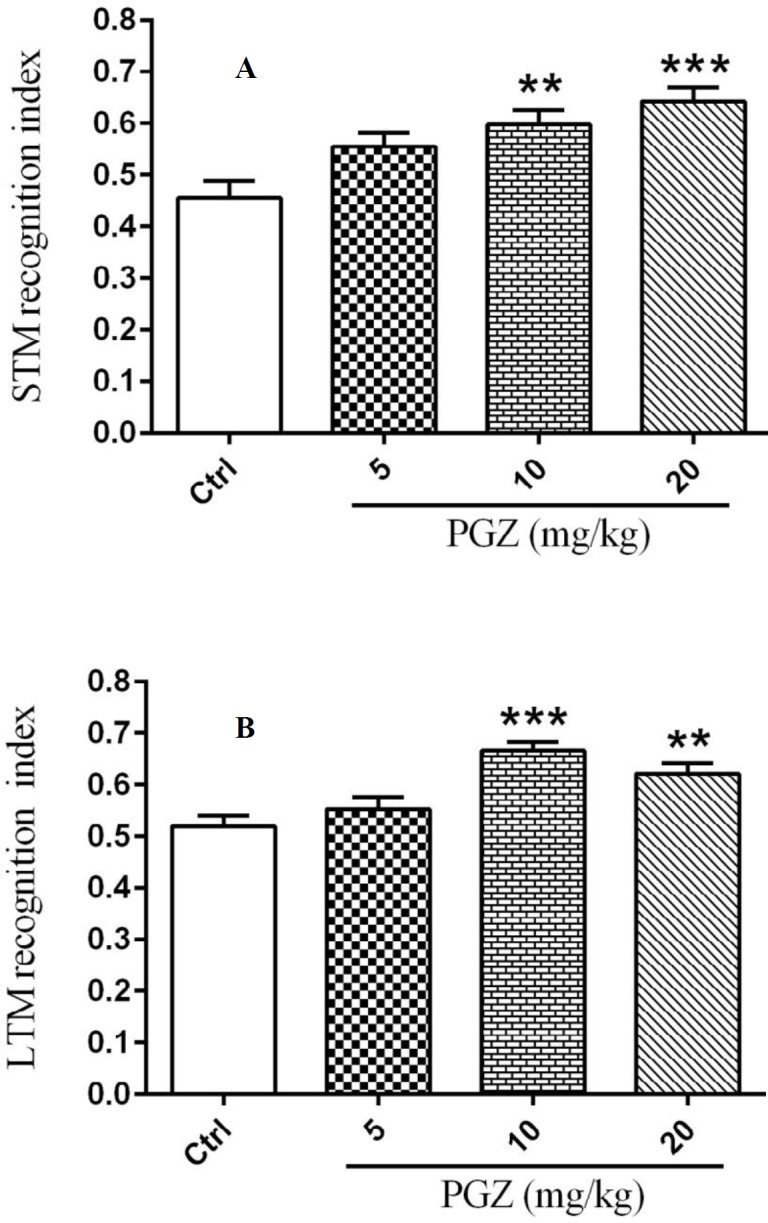
Effect of PGZ on cognitive deficits (A and B) of rat pups following FS. n=10. **:* P*<0.01 and ***: *P*<0.001 different from control group. STM: Short-term memory; LTM: Long-term memory; Ctrl: Control; PGZ: Pioglitazone

**Figure 6:**
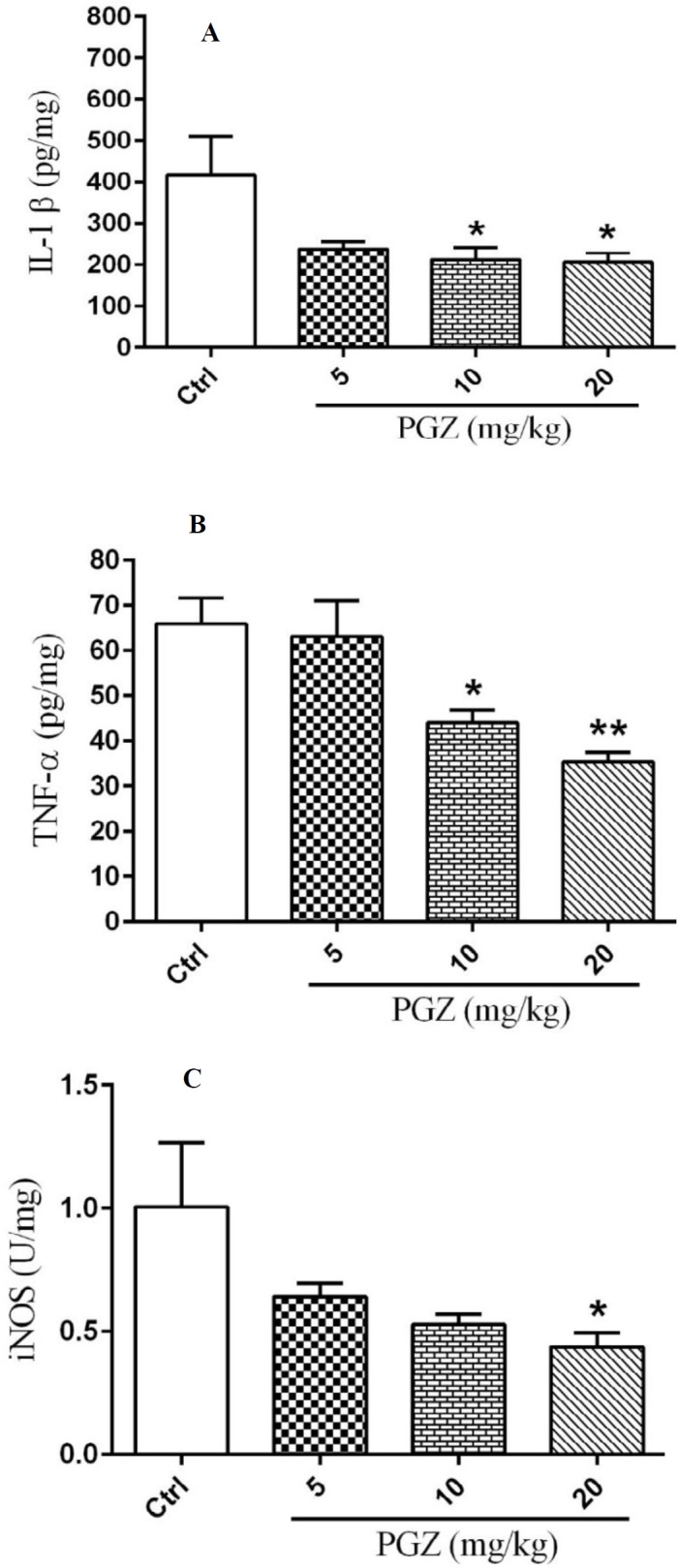
Effect of PGZ on IL-1β levels (n=7. *: *P*<0.05) (A), TNF-α levels (n=7. *: *P*<0.05 and **: *P*<0.01) (B) and iNOS levels (n= 7. *: *P*<0.05) (C) in the hippocampus of rat pups following FS, different from control group. IL-β: Interleukin 1 beta; TNF-α: Tumor necrosis factor-alpha; iNOS: Inducible nitric oxide synthase; Ctrl: Control; PGZ: Pioglitazone

**Figure 7:**
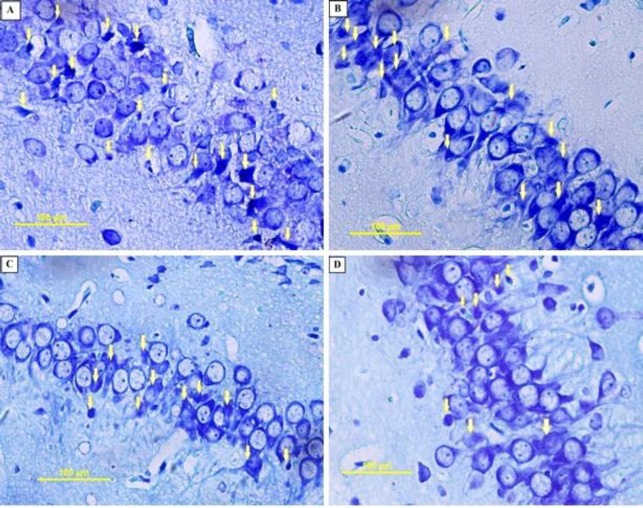
Photomicrographs of the rat pups’ hippocampus. PGZ decreased the degenerating neurons in the CA1 subfield of the hippocampus following FS. A: Ctrl group, B: PGZ (5 mg/kg) group, C: PGZ (10 mg/kg) group and D: PGZ (20 mg/kg) group. Arrowheads point to representative degenerating neurons. Scale bars: 100 µm. PGZ: Pioglitazone

**Figure 8:**
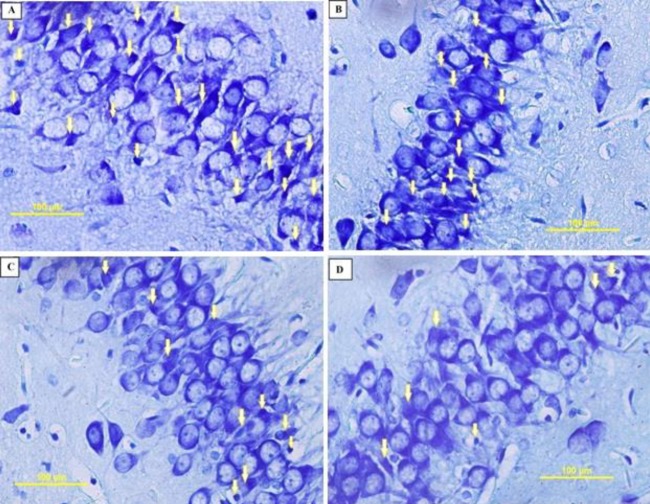
Photomicrographs of the rat pups’ hippocampus. PGZ decreased the degenerating neurons in the CA3 subfield of the hippocampus following FS. A: Ctrl group, B: PGZ (5 mg/kg) group, C: PGZ (10 mg/kg) group and D: PGZ (20 mg/kg) group. Arrowheads point to representative degenerating neurons. Scale bars: 100 µm. PGZ: Pioglitazone

**Figure 9:**
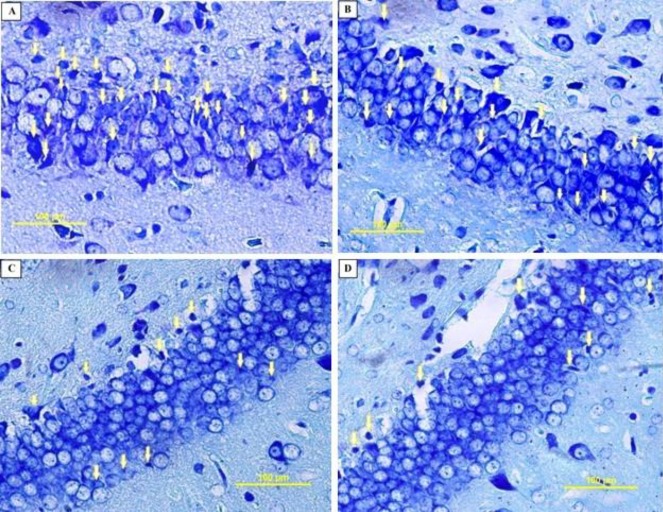
Photomicrographs of the rat pups’ hippocampus. PGZ decreased the degenerating neurons in the DG subfield of the hippocampus following FS. A: Ctrl group, B: PGZ (5 mg/kg) group, C: PGZ (10 mg/kg) group and D: PGZ (20 mg/kg) group. Arrowheads point to representative degenerating neurons. Scale bars: 100 µm. PGZ: Pioglitazone

**Figure 10:**
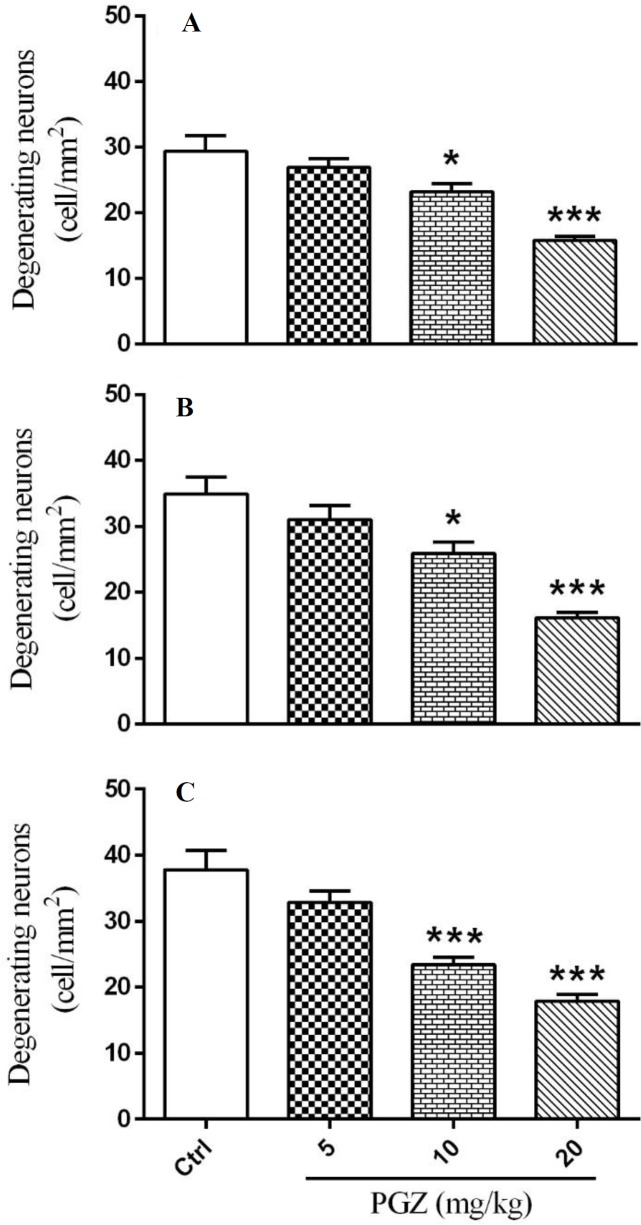
Effect of PGZ on degenerating neurons in CA1 (A), CA3 (B), and DG (C) regions of the hippocampus of rat pups following FS. n= 7. *: *P*<0.05 and ***: *P*<0.001 different from control group. Ctrl: Control; PGZ: Pioglitazone

**Figure 11:**
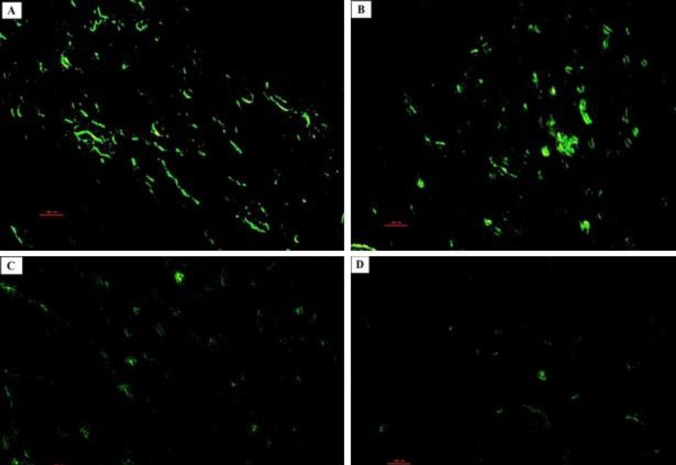
Photomicrographs of the rat pups’ hippocampus. PGZ decreased the TUNEL positive neurons in the CA1 subfield of the hippocampus following FS. A: Ctrl group, B: PGZ (5 mg/kg) group, C: PGZ (10 mg/kg) group and D: PGZ (20 mg/kg) group. Scale bars: 100 µm. PGZ: Pioglitazone

**Figure 12:**
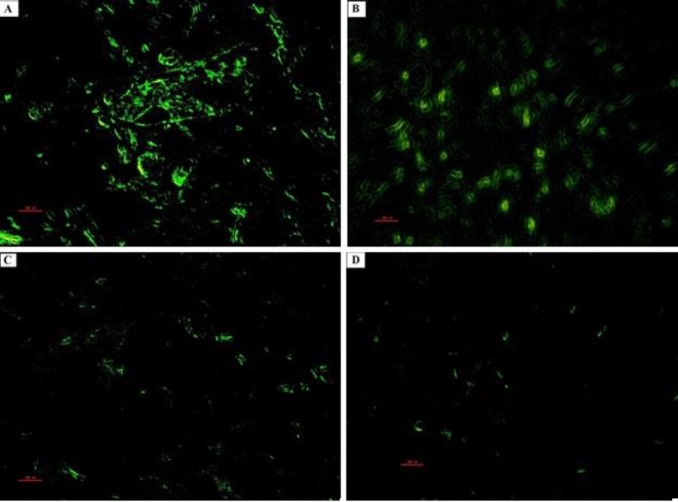
Photomicrographs of the rat pups’ hippocampus. PGZ decreased the TUNEL positive neurons in the CA3 subfield of the hippocampus following FS. A: Ctrl group, B: PGZ (5 mg/kg) group, C: PGZ (10 mg/kg) group and D: PGZ (20 mg/kg) group. Scale bars: 100 µm. PGZ: Pioglitazone

**Figure 13:**
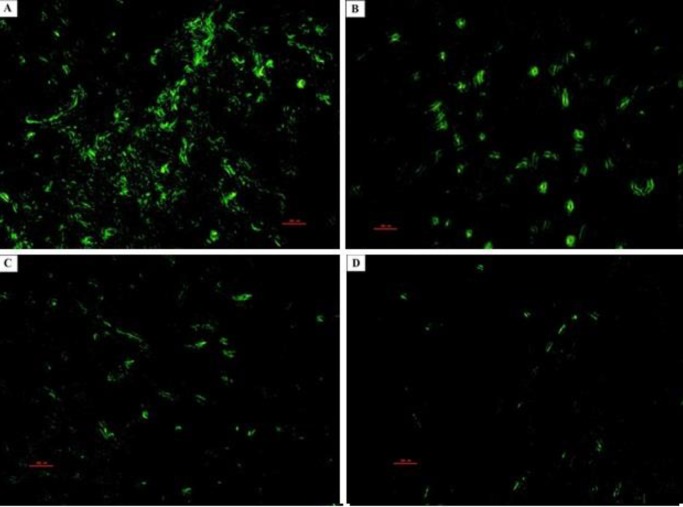
Photomicrographs of the rat pups’ hippocampus. PGZ decreased the TUNEL positive neurons in the DG subfield of the hippocampus following FS. A: Ctrl group, B: PGZ (5 mg/kg) group, C: PGZ (10 mg/kg) group and D: PGZ (20 mg/kg) group. Scale bars: 100 µm. PGZ: Pioglitazone

**Figure 14:**
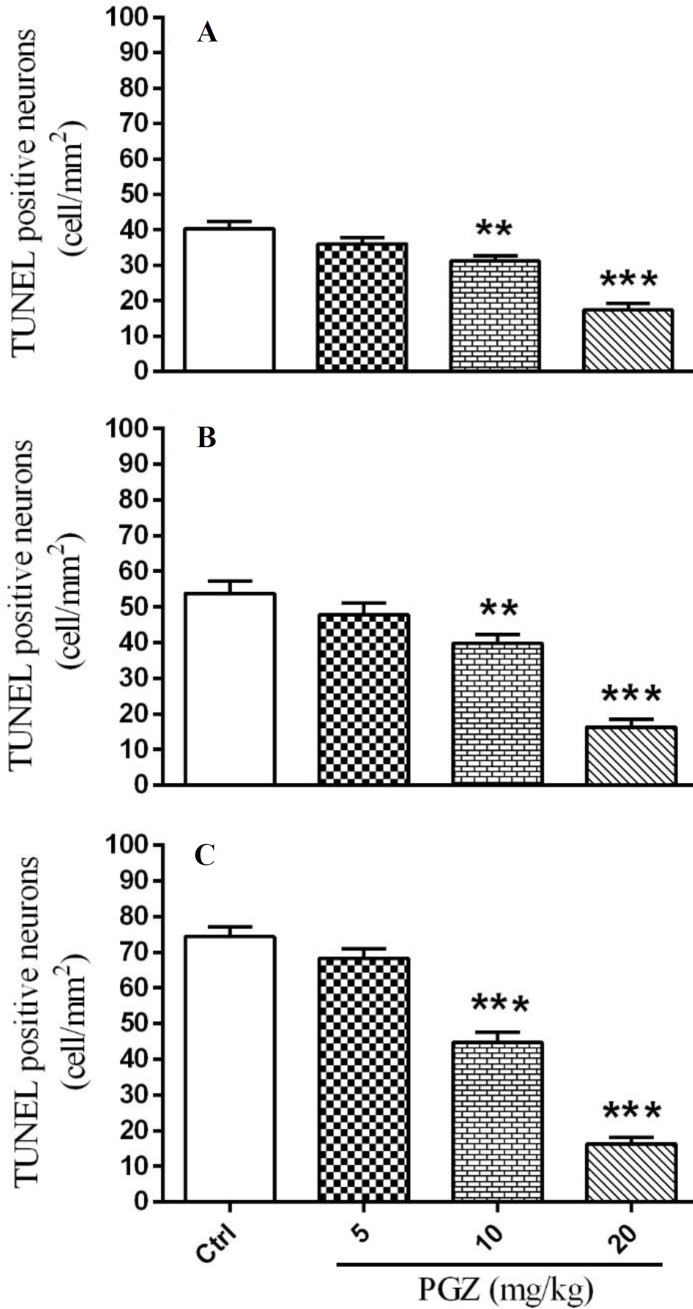
Effect of PGZ on TUNEL positive neurons in CA1 (A), CA3 (B), and DG (C) regions of the hippocampus of rat pups following FS.

n= 7. **: *P*<0.01 and ***: *P*<0.001 different from control group. Ctrl:

Control; PGZ: Pioglitazone

***Novel object recognition task (NORT)***

A typical apparatus for NORT is a 32 × 52 × 30 cm box made of Plexiglas (30). This test consists of a habituation phase, which is followed by a familiarization phase. During the habituation phase, at day 18 (P18), each rat pup was allowed to explore the empty arena freely for 3 days. Habituation consisted of three 10-min sessions per day. During familiarization trials, at day 21 (P21), rat pups were exposed to two identical objects (A1 and A2) (30). The objects were positioned in two adjacent corners, 9 cm away from the walls (31). In the test trials, animals were exposed to a familiar object (A1) and two novel objects that were similar in texture, color, and size, but had distinctive shapes (B and C). Short-term memory (STM) retention test trial was given 1.5 hr. after the familiarization session; rat pups were allowed to explore the open field for 5 min in the presence of two objects: the familiar object (A1) and a novel object (B). These objects were placed in the same locations as in the familiarization trial (32). Long-term memory (LTM) retention test trial was carried out 24 hrs. after the familiarization trial; at day 22 (P22), rat pups were allowed to explore the open field for 5 min in the presence of the familiar object (A1) and a third novel object (C) (32).

Object exploration was measured using two stopwatches to record the time spent exploring the objects during the experimental sessions. Exploration was defined as sniffing or touching the object with the nose. Sitting on the object was not considered exploration. A recognition index for each animal was calculated as follows: [TN/(TF + TN)], in which TF = time spent exploring the familiar object (A1) and TN = time spent exploring the novel object (B or C) (33).

***Enzyme-linked immunosorbent assay (ELISA)***

To prepare the tissue for the ELISA assay, the hippocampi were collected, frozen, and stored at -80 ^°^C until the measurement of IL-1β, TNF-α, and iNOS (34).

For analysis, hippocampi were homogenized. After that, samples derived from homogenization were centrifuged at 4500 x g for 15 min. Supernatants were collected and immediately stored at -80 ^°^C. Levels of IL-1β, TNF-α and iNOS were measured with ELISA kits according to the manufacturer’s instructions (35, 36). Total protein content was measured in each sample using the Bradford assay (37). Data were expressed as pg/mg or U/mg of total protein.

***Toluidine blue staining***

24 hrs. after the experiments, the rat pups were deeply anesthetized and brain samples were removed carefully, washed with sterile normal saline, and fixed in 10% neutral buffered formalin for 5 days at ambient temperature. After fixation, the samples were dehydrated with ascending ethanol series, cleared with xylene, and embedded in paraffin. The paraffin blocks were cut into 10 µm coronal sections at the level of the dorsal hippocampus (1–3 mm posterior from bregma) using a microtome. Six sections from each brain at 150 µm intervals were collected and stained with toluidine blue (38, 39).

All slides were observed with a light microscope (Olympus BX51, Japan) using a 40 x objective lens. Images were captured digitally from different subfields of the hippocampus including CA1, CA3, and DG of both hemispheres (40).

***TUNEL assay***

Paraffin-embedded brain sections of 10 µm were used for evaluation of apoptosis. An *in situ* cell death detection kit (fluorescein) was used to determine apoptotic neurons in the hippocampus according to the manufacturer’s protocol (41).

First, tissue samples were fixed in 10% neutral buffered formalin for 24 hrs. and embedded in paraffin. Sections were adhered to glass slides pretreated with 0.01% aqueous solution of poly-L-lysine then air dried. Tissue sections were deparaffinized by heating the slides for 30 min at 60 ^°^C followed by 2×5 min incubations in a xylene bath at room temperature, rehydrated by transferring the slides through a graded ethanol series: 3 min 100% ethanol, 3 min 95% ethanol, 3 min 90% ethanol, 3 min 80% ethanol, 3 min 70% ethanol, 3 min double-distilled water, and 3 min PBS (42).

Then, sections were treated with 20 μg/ml proteinase K for 20 min at room temperature. The sections were treated with 3% H_2_O_2_ in methanol for 10 min to inactivate endogenous peroxidase. After washing with PBS, sections were permeabilized by adding the permeabilization solution (0.1% Triton X-100 + 0.1% sodium citrate). After that, sections were washed again in PBS, incubated in the labeling reaction mixture containing terminal deoxynucleotidyl transferase and the deoxynucleotide for 1 hr. at 37 °C, avoiding exposure to light. Tissue sections were washed 2×5 min in PBS then air dried; they were covered with coverslips using antifade mounting medium. TUNEL positive neurons were detected under a fluorescence microscope (Olympus BX51, Japan). Images were captured from different subfields of the hippocampus including CA1, CA3, and DG of both hemispheres (43, 44).

***Counting of degenerating and TUNEL positive neurons***

For counting of degenerating and TUNEL positive neurons per unit area of the CA1, CA3, and DG subfields of the hippocampus, the morphometric method was used. All selected sections were digitally photographed and the number of degenerating neurons and TUNEL positive neurons were computed by a 10000 μm^2^ counting frame. The mean number of neurons (N_A_) in different subfields of the hippocampus was calculated using the following formula: N_A_= ΣǬ/(a/f×ΣP)

In the mentioned formula, “ΣǬ” is the summation of counted neurons that appeared in the sections, “a/f” is the area associated with each frame (10000 μm^2^), “ΣP” is the summation of frame-associated points hitting the reference (45, 46). 

***Statistical analysis***

The data are expressed as means±SEM except for seizure severity, which was represented as median ± interquartile. The GraphPad Prism 6.0 software (GraphPad Software Inc., USA, version 6) was used for statistical analysis. Comparisons among different groups were performed using one-way analysis of variance (ANOVA) followed by Tukey’s test as post-test. The data that were not normally distributed (seizure severity) were analyzed using non-parametric tests. Differences were considered statistically significant when *P*<0.05.

## Results

***Behavioral evaluation of febrile seizure***

Seizure latency, duration, and severity were used to assess the anticonvulsant effect of PGZ on FS. Rat pups were monitored for 1 hr after induction of FS. Wet-dog shakes were the first seizure-related behavior which rated as stage 1. While generalized seizures and rearing were rated as stage 3. Latencies for both stage 1 and 3 of FS were examined. Also, durations of previous stages of FS were recorded. 

Results showed that PGZ at doses of 10 and 20 mg/kg significantly increased stage 1 and 3 latencies at both 2^nd^ and 3^rd^ FS (Figure 2A, 2B, *P*<0.05, *P*<0.01 and *P*<0.001). Also, PGZ (10 and 20 mg/kg) significantly decreased stage one and three durations (Figure 2C, 2D, *P*<0.05 and *P*<0.001).

In addition, PGZ (10 and 20 mg/kg) significantly reduced the median of seizure scores (Figure 3, *P*< 0.001). So, the rat pups that were treated with PGZ showed less seizure-related behaviors than their control group.

***Body temperature***

The body temperature of rat pups was monitored after induction of FS. One-way ANOVA demonstrated no significant difference between groups after 1^st^ FS. However, PGZ significantly lowered body temperature when compared to the control group after 2^nd^ and 3^rd^ FS (Figure 4, *P*<0.05, *P*<0.01 and *P*<0.001).

***NORT***

NORT was used to evaluate the effect of PGZ on cognitive deficits following FS in rat pups. One-way ANOVA demonstrated significant effects of PGZ on STM and LTM recognition indexes compared to the control group (Figure 5, *P*<0.01 and *P*<0.001). This means that PGZ-treated rats had better short- and long-term memory performances than their control group.

***ELISA***

The results demonstrated that PGZ at the doses of 10 and 20 mg/kg significantly reduced IL-1β and TNF-α levels compared to the control group (Figure 6A, 6B, *P*<0.05 and *P*<0.01, respectively). Moreover, PGZ decreased the iNOS level at the dose of 20 mg/kg (Figure 6C, *P*<0.05 and *P*<0.01). 

***Apoptosis***

We measured the number of degenerating and TUNEL positive neurons in different subfields of the hippocampus. Results showed that PGZ (10 and 20 mg/kg) reduced the number of degenerating neurons in the CA1, CA3, and DG subfields (Figures 7, 8, 9, and 10, respectively, *P*<0.05 and *P*<0.001) in comparison with the control group. Also, PGZ (10 and 20 mg/kg) diminished the number of TUNEL positive neurons in the CA1, CA3, and DG subfields (Figures 11, 12, 13, and 14, respectively, *P*<0.01 and *P*<0.001).

## Discussion

Our findings revealed the ability of PGZ to ameliorate seizure severity and cognitive deficits induced by FS in rat pups. In addition, PGZ reduced inflammation and apoptosis in the hippocampus. 

Frequent seizures, during brain development, may provoke impairment of learning and memory (47, 48). Also, they may lead to sustained dysfunction of the hippocampal cells even in the absence of neuronal damage (49). FS can change the hippocampal expression of both Bcl2 and Bax proteins, resulting in apoptosis of neuronal cells in the hippocampus (50). In addition, early-life inflammation with FS may lead to long-lasting molecular changes and increased excitability in the adult rat hippocampus (51). Pro-inflammatory cytokines have been reported to be elevated in the developing brains exposed to FS. FS, by triggering inflammation, may enhance rapid kindling epileptogenesis in the immature rat brain (52). So, finding anti-inflammatory drugs for preventing FS and subsequent epileptogenesis and cognition dysfunction has been explored (53, 54). As mentioned, we found that PGZ protected rat pups against febrile seizures. PGZ increased seizure latency and decreased seizure duration and severity after induction of FS. In accordance with our finding, Adabi Mohazab *et al.* (2012) and Okada *et al.* (2006) in their studies demonstrated that PGZ exhibited anticonvulsant effects through activation of PPAR-γ in two different experimental models of seizure (55, 56). It was demonstrated that PGZ, via enhancement of PPAR-**γ** expression, prolonged the latency to flurothyl-induced seizures (57). Furthermore, PGZ was able to improve the anti-seizure effect of ketogenic diet against flurothyl-induced seizures in mice (57). Thus, it may be suggested that PGZ, by activation and expression of PPAR-**γ,** has the potential to reduce seizures. 

There are reports showing that repeated febrile seizures in children can affect their recognition memory (58). To investigate the effect of FS on memory, we used NORT to assess cognitive deficits. STM and LTM indexes were increased in animals pre-treated with PGZ. Therefore, it may be suggested that PGZ enhanced cognitive performance in rats that were exposed to FS. In line with the present findings, Jiang *et al.* (2012) and Yin *et al.* (2013) showed that PGZ reversed memory impairment in rats via various mechanisms including activation of PPAR-**γ**, inhibition of inflammation, and improvement in antioxidant defense system (59, 60). 

In spite of the induction of FS, we detected a decrease in body temperature of rat pups treated with PGZ after 2^nd^ and 3^rd^ febrile seizures. Considering the important role of cytokines in the development of body temperature (61, 62), we speculated that change in the levels of the main pro- and anti-inflammatory cytokines might be responsible for this effect of PGZ. The results showed that PGZ reduced IL-1β, TNF-α, and iNOS levels in the hippocampus of rats indicating a significant anti-inflammatory effect. Fever is a physiological response and mostly cytokine-mediated (63). Therefore, it may be suggested that PGZ, via attenuation of inflammation, reduced body temperature of rats. Similar to present findings, it was reported that PGZ attenuated inflammation in mice via activation of PPAR-γ receptor and suppression of IL-1β and TNF-α expressions (22, 24). Moreover, it was demonstrated that PGZ has a regulatory role in inflammation via inhibiting iNOS expression and NO generation (21, 23). The anti-inflammatory effect of pioglitazone has been implicated in other experimental models including the septic shock (22), nephropathy (64), atherosclerosis (65), and multiple sclerosis (66). 

The hippocampus is a brain region with a major role in the formation and recall of memories (67). Previous studies show that children with recurrent febrile seizures may have memory dysfunction that has a reverse association with their hippocampi size (58). So, we chose the hippocampus for more histological evaluation. In the present study, administration of a sub-convulsive dose of KA induced apoptosis. This finding is in accordance with the results of Lee and colleagues (67). This effect may be due to the net effect of KA on apoptosis or the combinational effect of both KA and LPS. We showed that PGZ attenuated the number of degenerating and TUNEL positive neurons in the hippocampus of rat pups exposed to FS. This finding implies that PGZ exhibited an anti-apoptotic effect in the hippocampus of rats. Similar to the present results, Lee *et al.* (2015) and Sauerbeck* et al.* (2011) in their studies showed that PGZ promoted a neuroprotective effect against KA-induced excitotoxicity due to attenuation of the activation of astrocytes and microglia (68, 69). Reductions of cytosolic cytochrome c and the key downstream executioner caspase-3 have also been reported as other anti-apoptotic mechanisms of PGZ (70). Similarly, it was reported that activation of PPAR-γ ameliorated KA-induced neuronal cell death in the hippocampus via reducing the mitochondrial dysfunction, hindering the translocation of Bax and cytochrome c, and DNA fragmentation (71). So, this effect of PGZ may justify our finding that shows PGZ reversed the memory impairment induced by FS. At present, the main drugs that are used in the treatment of FS are diazepam and phenobarbital. However, the rate of adverse effects for these drugs has been reported up to 30% (72). PGZ is a low-cost antidiabetic drug with a very low chance of hypoglycemia when used as a monotherapy (73). Also, the anti-apoptotic and anti-inflammatory effects of PGZ in the brain make it a potential candidate to treat febrile seizure and its consequences including cognitive dysfunction. However, much more study is needed to justify such application. 

## Conclusion

The present results revealed the ability of PGZ to ameliorate febrile seizures and cognitive deficits through anti-apoptotic and anti-inflammatory mechanisms in the hippocampus of rat pups following febrile seizure.

## Conflicts of Interest

The authors declare no conflicts of interest.
